# Direct evidence of boosted oxygen evolution over perovskite by enhanced lattice oxygen participation

**DOI:** 10.1038/s41467-020-15873-x

**Published:** 2020-04-24

**Authors:** Yangli Pan, Xiaomin Xu, Yijun Zhong, Lei Ge, Yubo Chen, Jean-Pierre Marcel Veder, Daqin Guan, Ryan O’Hayre, Mengran Li, Guoxiong Wang, Hao Wang, Wei Zhou, Zongping Shao

**Affiliations:** 1 Centre for Future Materials, University of Southern Queensland, Springfield Central, Ipswich, QLD 4300 Australia; 2 WA School of Mines: Minerals, Energy and Chemical Engineering (WASM-MECE), Curtin University, Perth, WA 6102 Australia; 3 School of Materials Science and Engineering, Nanyang Technological University, 50 Nanyang Avenue, Singapore, 639798 Singapore; 4 John de Laeter Centre, Curtin University, Perth, WA 6102 Australia; 5 State Key Laboratory of Materials-Oriented Chemical Engineering, College of Chemical Engineering, Nanjing Tech University (NanjingTech), Nanjing, 210009 P. R. China; 6 Department of Metallurgical and Materials Engineering, Colorado School of Mines, Golden CO, 80401 USA; 7 School of Chemical Engineering, The University of Queensland, St. Lucia, QLD 4072 Australia

**Keywords:** Catalytic mechanisms, Solid-state chemistry, Materials chemistry, Electrocatalysis

## Abstract

The development of oxygen evolution reaction (OER) electrocatalysts remains a major challenge that requires significant advances in both mechanistic understanding and material design. Recent studies show that oxygen from the perovskite oxide lattice could participate in the OER via a lattice oxygen-mediated mechanism, providing possibilities for the development of alternative electrocatalysts that could overcome the scaling relations-induced limitations found in conventional catalysts utilizing the adsorbate evolution mechanism. Here we distinguish the extent to which the participation of lattice oxygen can contribute to the OER through the rational design of a model system of silicon-incorporated strontium cobaltite perovskite electrocatalysts with similar surface transition metal properties yet different oxygen diffusion rates. The as-derived silicon-incorporated perovskite exhibits a 12.8-fold increase in oxygen diffusivity, which matches well with the 10-fold improvement of intrinsic OER activity, suggesting that the observed activity increase is dominantly a result of the enhanced lattice oxygen participation.

## Abstract (web summary)

While water splitting provides a renewable means to store energy, the sluggish O_2_ evolution half-reaction limits applications. Here, authors examine a silicon-incorporated strontium cobaltite perovskite and correlate lattice oxygen participation in O_2_ evolution to the oxygen ion diffusivity.

## Introduction

In the societal pursuit of a sustainable energy future, the electrolysis of small molecules including water, dinitrogen and carbon dioxide is envisioned to play an important role, because it is central to the conversion of electrical energy, which can come from the vastly available renewable energies (e.g., solar and wind), into chemical energy stored in a range of fuels or chemicals such as hydrogen, ammonia and carbon monoxide^1–3^. While the kinetics for the reduction of these molecules determines the reaction rate, the overall electrical-to-chemical power conversion efficiency of these electrolytic processes is largely dependent on the anodic oxygen evolution reaction (OER), which provides electrons for the reduction reaction to occur but suffers from slow reaction kinetics associated with its four electron transfers. To date, iridium- and ruthenium-based materials are among the best-performing OER catalysts in aqueous solutions^4^. However, the scarcity and prohibitive cost of Ir and Ru pose major obstacles towards widespread use in electrolysis technologies. These concerns have encouraged tremendous research activities in finding efficient and low-cost alternatives, among which nonprecious transition metal oxides featuring a perovskite structure have been demonstrated excellent OER activities. Indeed, the best perovskites show performance comparable to (if not higher than) Ir-/Ru-based standards, especially in alkaline media^5,6^. Typical examples include single perovskite Ba_0.5_Sr_0.5_Co_0.8_Fe_0.2_O_3−*δ*_ (BSCF) and double perovskite PrBaCo_2_O_5+*δ*_ (PBC), which have both been shown with exceptional intrinsic activity in alkaline electrolytes, in some cases several orders of magnitude higher than the IrO_2_ benchmark^7–13^.

Over the past years, our understanding of the OER mechanism has proven to be instrumental in developing better catalysts. Taking the OER on perovskite surfaces for an example, the conventional adsorbate evolution mechanism (AEM) proceeds via a sequence of concerted electron–proton transfers on the transition metal active centres^7^, whose binding to the adsorbed oxygen intermediates should be neither too strong nor too weak to achieve optimal activity according to the Sabatier’s principle^14^. This has initiated the exploration of electronic structure parameters that can serve as activity descriptors to help screen highly efficient catalyst candidates^7,15–17^. For instance, the filling of the 3d electron with an *e*_g_ symmetry of surface transition metal cations has been successfully utilised to identify several state-of-the-art perovskite OER catalysts such as BSCF^7^, CaMnO_2.5_^18^ and SrNb_0.1_Co_0.7_Fe_0.2_O_3–*δ*_ (SNCF)^19^. However, the performance of oxide electrocatalysts based on AEM is limited by the scaling relations between the oxygen intermediates, which, according to Man et al.’s^20^ density functional theory (DFT) calculations, can lead to a considerable overpotential for the OER.

More recently, a new mechanism based on the redox chemistry of lattice oxygen anions has been proposed. Often termed the lattice oxygen-mediated mechanism or lattice oxygen oxidation mechanism (LOM), this mechanism involves the direct participation of oxygen anions from the perovskite lattice as an active intermediate in the OER, which was supported by ^18^O isotope detection of the reaction product as well as DFT calculations^21–26^. Of note, the LOM can also occur for other types of oxygen-containing OER catalysts, for example, Co–phosphate^27^, Co–Ni spinel oxide^28^ and Co–Zn oxyhydroxide^29^. Importantly, it is expected that a catalyst utilizing the LOM can bypass the limitations inherent in AEM-based catalysts where scaling relations constrain performance^20^, thereby potentially offering much improved OER activity^23^. This possibility accentuates the need to develop novel catalyst candidates that operate via the LOM pathway. Even more importantly, the degree to which the lattice oxygen participation could promote the OER activity for perovskite oxides is still unclear and must be explored.

It is well known that the perovskite structure is highly flexible and can therefore accommodate a wide variety of elements in the periodic table. For this reason, elemental doping has been extensively applied to the development of perovskite oxides for diverse fields of research, including OER electrocatalysis. However, since many of the transition metals are active towards the OER^30^, their incorporation into the perovskite structure may mask the real contribution of lattice oxygen participation in enhancing the catalytic activity. In addition, synergy could be created between the dopant and the parent cation in the perovskite^31^, which causes additional difficulty in distinguishing the contribution of lattice oxygen participation to the OER activity.

It was reported that a minor amount of silicon (Si) doping can stabilise the oxygen vacancy disordered cubic perovskite structure, thus significantly improving the oxide ion conductivity/oxygen ion diffusion rate as well as modifying the oxygen vacancy concentration^32,33^. Due to the much smaller size of Si^4+^ (*r* = 0.26 Å with a preferable tetrahedral coordination) than most of the B-site cations^34^, the solubility of Si in ambient-pressure synthesised perovskites is usually quite low (3–15% of the B-site), while the silicon-containing impurity phase is an insulator, which usually stays at the grain boundary and acts as an inhibitor for charge transfer^35^. By tailoring the amount of Si to be incorporated, materials with different oxygen vacancy concentrations and oxygen diffusion rates can be developed. Furthermore, Si by itself is inert towards electrocatalysis, hence the introduction of Si will not contribute additional catalysis towards the OER. Thus, Si incorporation may provide an excellent platform for investigating the role of lattice oxygen participation in the OER.

Strontium cobaltite, i.e., SrCoO_3–*δ*_ (SCO), is demonstrated both theoretically and experimentally with high OER activity, involving likely the operation of the LOM-type reaction mechanism^20,23–25^. In this study, we select silicon as a modifier for SCO to create several Si-incorporated SCO samples with different levels of oxygen diffusion rates and oxygen vacancy concentrations but similar surface transition metal properties, which are then applied as electrocatalysts to explore the role and degree of lattice oxygen participation in the OER process. pH-dependent OER kinetic studies and surface amorphization observations suggest that the LOM mechanism is operational during the OER on both SCO and Si-doped SCO. Notably, we achieve up to an order of magnitude higher OER intrinsic activity upon the inclusion of Si into the SCO lattice, approaching the activity of the benchmark BSCF, although the *e*_g_ filling of the former is far from ideal based on the AEM. This activity improvement matches closely with the 12.8-fold enhancement in the oxygen mobility. We therefore can strongly support the important role of LOM in substantially contributing to the OER activity. Our work opens an avenue to develop lattice-oxygen-participated catalysts towards efficient water oxidation for potential electrolysis applications.

## Results

### Structural characterisations

**Fig. 1:**
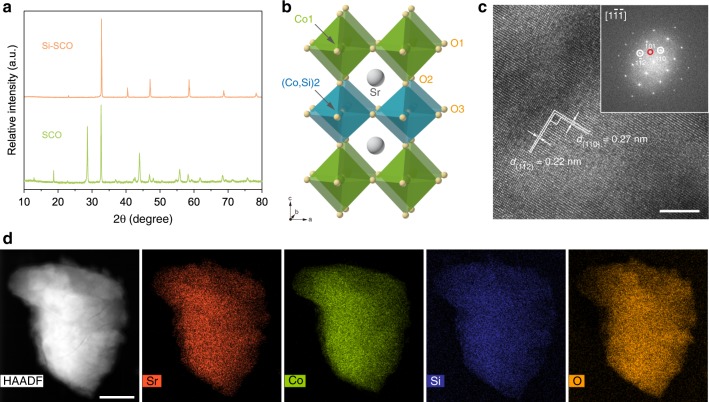
Structural characterisations of Si-incorporated strontium cobaltites. **a** XRD patterns showing the formation of a tetragonal phase upon the incorporation of Si into SCO. **b** A schematic depicting the tetragonal crystal structure of Si–SCO. **c** HRTEM image of Si–SCO and the corresponding FFT pattern with zone axis of [$1\bar{1}\bar{1}$]. **d** HAADF-STEM image of Si–SCO and the corresponding EDS mapping images of Sr, Co, Si and O. Scale bar in **c** is 5 nm and in **d** is 200 nm.

We first comparatively studied the pristine SCO and the Si-incorporated SCO with an intentional doping amount of 5% at the B-site (i.e., *y* = 0.05 in SrCo_1–*y*_Si_*y*_O_3–*δ*_, denoted as Si–SCO). Both samples were synthesised by a ball-milling-assisted solid-state reaction method (Methods section). X-ray diffraction (XRD) pattern as shown in Fig. 1a and the corresponding Rietveld refinement analysis (Supplementary Fig. 1a and Table 1) suggest that the parent SCO perovskite consists of a major Sr_6_Co_5_O_15_ phase and a small quantity of Co_3_O_4_ impurity, in line with previously reported results^36^. Incorporating Si into the B-site gave rise to the formation of a tetragonal phase, which has a space group of *P*4/*mmm* and lattice parameters of *a* = *a*_*p*_ ≈ 3.85917(4) Å and *c* ≈ 2*a*_*p*_ = 7.7270(1) Å (*a*_*p*_ being the lattice parameter of an ideal cubic-phase single perovskite with space group of *Pm*$\overline{3}$*m*) (Supplementary Fig. 1b). Two impurity phases, i.e., brownmillerite Sr_2_Co_2_O_5_ and monoclinic Sr_2_SiO_4_, were also detected in the Si–SCO sample with only minor weight fractions (Supplementary Table 2), based on which the nominal bulk composition of the major tetragonal phase was approximated to be Sr_0.98_Co_0.97_Si_0.03_O_3–*δ*_, where presence of A-site Sr-deficiency may be possible. A schematic illustration of this tetragonal structure is given in Fig. 1b, in which layers of Co1O_6_ and (Co,Si)2O_6_ octahedrons, both corner-shared, alternate with each other along the *c* axis [here Co1 and (Co,Si)2 refer to two different crystallographic positions at the B-site]. This doubling of the perovskite unit cell along the *c*-direction is characteristic of a double perovskite structure with B-site layered ordering^37^, resembling that of strontium cobaltites doped by main group VA elements such as phosphorus and antimony^38,39^. Figure 1c shows a high-resolution transmission electron microscopy (HR-TEM) image of Si–SCO, where lattice distances of 0.27 and 0.22 nm are observed, consistent with those of (110) and (1$\bar 1$2) planes calculated from the XRD data. In addition, the corresponding fast Fourier transformed (FFT) pattern further reveals the presence of cation-ordering reflections (marked by a red circle in Fig. 1c inset). Figure 1d displays high-angle annular dark-field scanning TEM (HAADF-STEM) and energy-dispersive X-ray spectroscopy (EDS) mapping images, demonstrating a homogeneous distribution of all the constituent elements of Sr, Co, Si and O. This confirms the incorporation of Si within the perovskite lattice and suggests that the impurity phases were evenly dispersed inside the oxide powder rather than separated as large aggregates. The overall morphology of SCO and Si–SCO was studied by scanning electron microscopy (SEM), as presented in Supplementary Fig. 2. Both samples show a large particle size in the (sub)micrometre range with no noticeable difference, except that Si–SCO has somewhat larger and more sintered particles compared with SCO. This is also supported by the relatively lower Brunauer–Emmett–Teller (BET) surface area of Si–SCO, approximately a quarter that of SCO (0.44 vs. 1.74 m^2^ g^−1^), as determined from multipoint Krypton (Kr) adsorption tests (Supplementary Table 3).

### Oxygen evolution activity

**Fig. 2:**
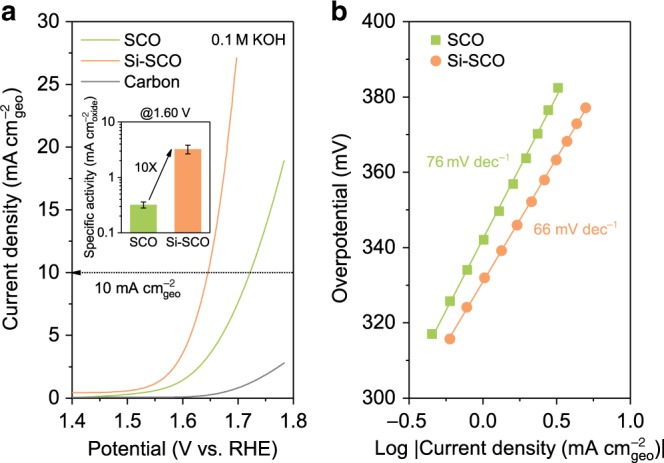
Electrocatalytic oxygen evolution performance of Si-incorporated strontium cobaltites. **a** OER kinetic currents (normalised to the geometric surface area of the electrode, in mA cm^−2^_geo_) of SCO and Si–SCO collected in an O_2_-saturated 0.1 M KOH electrolyte under ambient conditions. The contribution from the conductive carbon as catalyst support is shown for reference. Inset shows the OER specific activity (normalised to the BET surface area of the oxide catalyst, in mA cm^−2^_oxide_) of SCO and Si–SCO at 1.60 V vs. RHE. Error bars are the standard deviations of triplicate measurements. **b** Steady-state Tafel data for SCO and Si–SCO.

The electrocatalytic OER performance of SCO and Si–SCO was investigated using a rotating disk electrode (RDE) based three-electrode configuration under ambient conditions. To eliminate any contribution from the capacitive effect, cyclic voltammetry (CV) was performed in an O_2_-saturated 0.1 M KOH aqueous electrolyte at a 10 mV s^−1^ scan rate and at a 2000 rpm rotation speed, which were averaged and *iR*-corrected to obtain the OER kinetic currents (an example of this data processing can be found in Supplementary Fig. 3), as shown in Fig. 2a. As a common practice for evaluating perovskite oxide electrocatalysts (Supplementary Figs. 4 and 5, and Supplementary Note 1)^40^, the active materials were mixed at a mass ratio of 5:1 with conductive carbon, which facilitates electrical contact between catalyst particles as well as between the catalyst and the RDE while contributing negligibly to the OER currents (Fig. 2a). Compared with SCO, the kinetic current of Si–SCO markedly increases across the OER region, indicating a significantly enhanced OER activity. This is also the case when one compares the overpotential required to afford a geometric current density of 10 mA cm^−2^_geo_ (*η*_10_, a metric associated with solar fuel production^41^). Specifically, Si–SCO exhibits a *η*_10_ value of 417 mV, which is ~70 mV smaller than that of SCO (488 mV).

The catalyst surface area is known to influence the apparent OER activity observed on different catalysts. To assess this, we normalised the OER kinetic currents to the BET surface area of each perovskite catalyst (Supplementary Table 3), which allowed us to report the specific activity of the catalysts as a metric for comparing their intrinsic activity^7^. Figure 2a inset compares the specific activity at an applied potential of 1.60 V vs. the reversible hydrogen electrode (RHE), from which it is obvious that Si–SCO is intrinsically more active than SCO, showing a one order of magnitude higher specific activity. In addition, steady-state Tafel data suggest that Si–SCO gives a Tafel slope of 66 mV dec^−1^, lower than that of SCO (76 mV dec^−1^) (Fig. 2b). This is a good indication of the improved OER kinetics on the perovskite catalyst incorporating silicon because catalysts having a smaller Tafel slope tend to deliver significantly increased currents at only moderate increments of overpotential. A detailed comparison with literature results, as tabulated in Supplementary Table 4, suggests that the Si-incorporated Si–SCO catalyst compares favourably to the state-of-the-art perovskite catalysts such as BSCF, PBC and SNCF, among many others^7–13,18,19,31,42–47^.

### Oxygen evolution mechanism

**Fig. 3:**
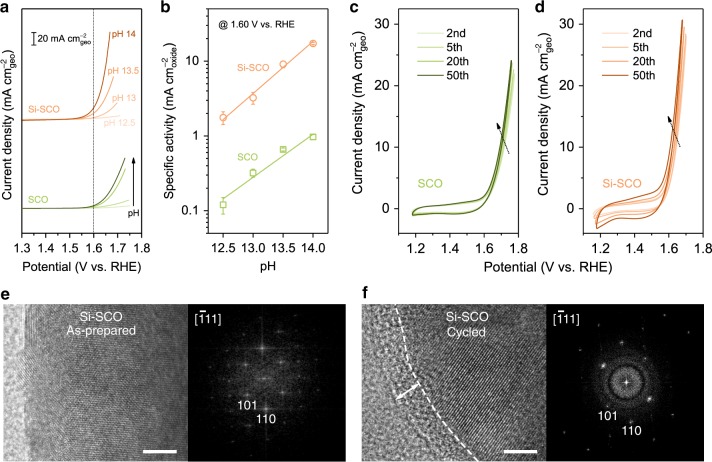
The oxygen evolution mechanism studies on Si-incorporated strontium cobaltites. **a** OER kinetic currents (in mA cm^−2^_geo_) of SCO and Si–SCO in O_2_-saturated KOH electrolytes with varying pH. **b** OER specific activity (in mA cm^−2^_oxide_) of SCO and Si–SCO at 1.60 V vs. RHE as a function of pH. **c**, **d** Select CV curves of **c** SCO and **d** Si–SCO in O_2_-saturated 0.1 M KOH over 50 cycles at a 10 mV s^−1^ scan rate. **e**, **f** HRTEM images and the corresponding FFT patterns of **e** as-prepared and **f** cycled (for 50 cycles) Si–SCO electrode. Scale bar in **e** and **f** is 5 nm.

Previous ^18^O-isotopic labelling experiments suggest that the OER on the pristine SCO can proceed via both the AEM and LOM pathways and that the LOM pathway plays an important role in delivering enhanced OER performance^25^. Specially, in alkaline electrolytes, the occurrence of LOM has been associated with the observation of pH-dependent OER kinetics on the RHE scale^25^, which can be deducted from Eq. (1)^48^:

1 $$i = \theta \cdot c_{{\mathrm{OH}}} \cdot {\mathrm{e}}^{ - \Delta G/RT}$$

where *i* is the OER current, *θ* is the surface coverage of the adsorbed hydroxide or oxyhydroxide intermediates, *c*_OH_ is the concentration of hydroxide ions, ∆*G* is the reaction free energy, *R* is the universal gas constant and *T* is the temperature during the measurement. Raising the pH can either modify the exponential term by altering the energy of the adsorbed intermediates or increase the pre-exponential term by increasing the surface coverage or the OH^−^ concentration, thus leading to increased OER activity. Consistent with this model, our experimental studies confirm an increase in the OER activity for both SCO and Si–SCO samples with increasing pH from 12.5 to 14 (Fig. 3a), indicative of pH-dependence of the OER kinetics and hence LOM participation. Figure 3b further compares the specific activity of both SCO and Si–SCO electrocatalysts at 1.60 V vs. RHE as a function of pH, from which the proton reaction orders on the RHE scale were extracted from the slopes [*ρ* = (∂log*i*/∂pH)_*E*_] to be 0.58 and 0.70 for SCO and Si–SCO, respectively, in accord with reported values for Co-based perovskite oxides^25,48^. These results strongly suggest that the LOM mechanism is likely at play during the OER on SCO and Si–SCO, agreeing well with the literature results concerning the lattice oxygen participation in the OER over SCO^25^.

Further evidence supporting the operation of the LOM mechanism can come from the observation of catalyst surface reconstruction during potential cycling, especially for the initial 50 cycles^49,50^. As shown in Fig. 3c, d, the pseudocapacitive and OER currents of SCO and Si–SCO were found to increase with the continuous CV cycling, indicative of the occurrence of surface amorphization, a phenomenon that was similarly observed for BSCF^26,51^. Of significance, this change appears to be more drastic for Si–SCO, which, in line with its higher proton reaction order, may suggest a higher tendency for its lattice oxygen to participate in the OER. The surface amorphization of Si–SCO was also confirmed by TEM investigations (Fig. 3e, f), in which an amorphous region of ≈5 nm was found on the cycled electrode in contrast to the largely crystalline surface of the as-prepared catalyst. These results further indicate the possible involvement of lattice oxygen redox during the OER on Si-incorporated Si–SCO, although it occurs at the expense of surface stability, which will be discussed later in more details.

### Origin of the improved OER activity for Si–SCO

To understand the activity enhancement, we investigated the changes in physicochemical properties induced by Si-incorporation. As mentioned earlier, for Si-doping under ambient-pressure conditions, Si enters the perovskite framework with four-fold coordination to the oxygen (i.e., in the form of orthosilicate SiO_4_^4−^)^32^. Therefore, the introduction of tetrahedral Si into the octahedral Co site can give rise to the generation of oxygen vacancies, which in turn results in a decrease in the bulk Co oxidation state, as can be seen from the below defect equation (Kröger–Vink notation):

2 $${\mathrm{SiO}}_{\mathrm{2}} + {\mathrm{2Co}}_{{\mathrm{Co}}}^ \times \to {\mathrm{Si}}_{{\mathrm{Co}}}^ \times + {\mathrm{2Co}}_{\mathrm{Co}}^\prime + {\mathrm{V}}_{\mathrm{O}}^{ \cdot \cdot } + {\mathrm{1}}/{\mathrm{2O}}_{\mathrm{2}} + {\mathrm{O}}_{\mathrm{O}}^ \times$$

**Fig. 4:**
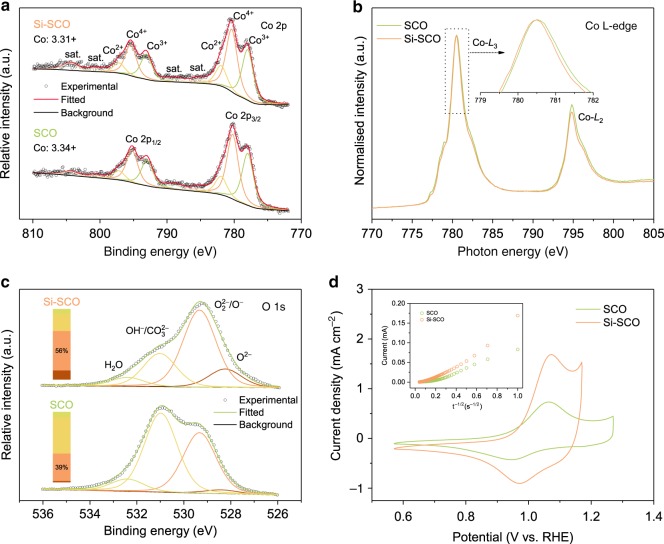
Chemical and electrochemical characterisations of Si-incorporated strontium cobaltites. **a** Co 2*p* core-level XPS spectra of SCO and Si–SCO, with peak fitting results based on multiple cobalt species. Here sat. denotes satellite peaks. **b** Co L-edge NEXAFS spectra of SCO and Si–SCO. Inset of **b** shows the Co L_3_-edge spectra in an expanded photon energy scale. **c** O 1*s* core-level XPS spectra of SCO and Si–SCO, with peak fitting results illustrated in the stacked columns. **d** CV curves of SCO and Si–SCO in Ar-saturated 6 M KOH, where redox peaks indicate the electrochemical oxygen intercalation/de-intercalation. Inset of **d** shows the chronoamperometry data (*i* vs. t^–1/2^) used for the calculation of oxygen ion diffusion coefficients.

This justifies the stabilisation of the Si-doped perovskite structure in which the effect of the smaller size of Si^4+^ is balanced by that of the larger size of reduced Co ions^34^. Indeed, results from iodometric titrations suggest that in the bulk of the Si^4+^-incorporated material an increase in the oxygen vacancy concentration occurred in concert with a reduction in the Co oxidation state (Supplementary Table 5). Specifically, Si–SCO exhibits a higher level of oxygen deficiencies relative to SCO (*δ* = 0.35 vs. 0.25), and concomitantly a lower average valence state of the bulk Co cations (3.32+ vs. 3.50+). However, regarding the surface chemical state of Co, which is of greater relevance because the OER takes place on the catalyst surface, we observed no obvious difference using surface-sensitive techniques of X-ray photoelectron spectroscopy (XPS) and near-edge X-ray absorption fine structure spectroscopy (NEXAFS), as shown in Fig. 4a, b. For example, only a very small shift toward lower photon energies was found at the Co L_3_-edge as Si^4+^ is incorporated into SCO (Fig. 4b), indicative of an insignificant decline in the surface Co valence^52^. Considering the inertness of silicon towards electrocatalysis, the OER activity of SCO and Si–SCO based on the AEM pathway should be primarily determined by the valence of B-site surface cobalt ions^7^. The little change in surface Co state thus suggests that the contribution of AEM to the OER activity can be reasonably considered unchanged after Si incorporation. Based on peak deconvolution analysis following an earlier report^53^, we estimate the surface Co oxidation state to be 3.34+ and 3.31+ for SCO and Si–SCO, respectively (Fig. 4a), which gives an approximate *e*_g_ filling number of 0.7 assuming that the Co cations are in the intermediate spin state. This value diverges significantly from *e*_g_ ≈ 1.2, as predicted for highly active perovskite catalysts based on the AEM mechanism (e.g., BSCF)^7^. It thus suggests that other than AEM, the operation of LOM likely contributes significantly to the high overall OER activity observed on the Si–SCO catalyst.

As for the surface oxygen state, an increase in the oxygen vacancy concentration was observed, consistent with that in the bulk. As shown in Fig. 4c and Supplementary Table 6, XPS results of the O 1 *s* core level, which were fitted by four components assigned as adsorbed water (H_2_O at ~532.4 eV), hydroxide or carbonate (OH^−^/CO_3_^2−^ at ~531.0 eV), oxidative oxygen (O_2_^2−^/O^−^ at ~529.4 eV) and lattice oxygen (O^2−^ at ~528.3 eV), show a larger number of O_2_^2−^/O^−^ species on Si–SCO than on SCO, suggesting a higher content of surface oxygen vacancies^54^. It is interesting to note that the large presence of surface hydroxide/carbonate is indicative of Sr segregation at the perovskite surface, a phenomenon commonly observed in Sr-containing perovskites prepared from conventional methods (e.g., solid-state reaction). However, the extent of Sr segregation does not vary significantly across different samples, and thus should not considerably affect the OER activity (Supplementary Fig. 6, Supplementary Tables 7 and 8, and Supplementary Note 2). To further corroborate the increased oxygen vacancy concentration through Si doping, the electrochemical oxygen intercalation in SCO and Si–SCO was probed through CV experiments conducted in an Ar-saturated 6 M KOH solution. As depicted in Fig. 4d, redox peaks appear as oxygen ions are inserted into and extracted from the accessible lattice vacancy sites (with an occupancy fraction of *σ*). This is associated with a pseudocapacitive-type intercalation process that can be represented by Eq. (3):

3 $${\mathrm{SrCo}}_{{\mathrm{1}}-y}{\mathrm{Si}}_y{\mathrm{O}}_{{\mathrm{3}}-\delta } + {\mathrm{2}}\sigma {\mathrm{OH}}^- \leftrightarrow {\mathrm{SrCo}}_{{\mathrm{1}}-y}{\mathrm{Si}}_y{\mathrm{O}}_{{\mathrm{3}}-\delta + \sigma } + \sigma {\mathrm{H}}_{\mathrm{2}}{\mathrm{O}} + {\mathrm{2}}\sigma {\mathrm{e}}^-$$

It is interesting to note that this oxygen intercalation is accompanied by the oxidation of Co when one considers the charge neutrality for SrCo_1–*y*_Si_*y*_O_3–*δ*_ (before oxygen intercalation) and SrCo_1–*y*_Si_*y*_O_3–*δ*+*σ*_ (after oxygen intercalation). Of importance, Si–SCO, having more vacant oxygen sites, displayed a larger current density in the intercalation regime, thereby signifying a greater propensity for oxygen intercalation^24^. Moreover, the increase in oxygen vacancy content resulted in positively shifted intercalation redox peaks in Si–SCO with respect to SCO, as can be elucidated by the pseudocapacitive Nernst Equation^55^:

4 $$E = E^0 + \left( {{\mathrm{RT}}/nF} \right){\mathrm{ln}}\left[ {\sigma /\left( {{\mathrm{1}}-\sigma } \right)} \right]$$

where *E* and *E*^0^ represent the measured and standard potential for oxygen intercalation, respectively, *n* is the number of electrons transferred and *F* is the Faraday constant.

Following the oxygen intercalation measurements, the oxygen ion diffusion coefficients (*D*_O_) of SCO and Si–SCO were determined using chronoamperometry with the results presented in the Fig. 4d inset, where current was plotted as a function of the inverse square root of time. By applying a bounded three-dimensional diffusion model reported earlier^24,56,57^ (Methods section), the *D*_O_ value of SCO at room temperature was calculated to be 0.94 × 10^−11^ cm^2^ s^−1^, a value that agrees with literature results for strontium cobaltites^58^. Remarkably, Si–SCO had a diffusion coefficient of *D*_O_ = 12.04 × 10^−11^ cm^2^ s^−1^, which is ~12.8 times faster than SCO, and correlates well with the 10-fold improvement in intrinsic OER activity. The accelerated oxygen ion diffusion is likely associated with the increased crystal lattice symmetry, i.e., from hexagonal symmetry for SCO to tetragonal symmetry for Si–SCO. Although the energy landscape through which the oxygen anions migrate remains quite complex, one general comment is that a higher symmetry could lead to faster oxygen anion transport^59–61^. Meanwhile, the fast oxygen ion diffusion in Si–SCO is believed to be related to its unique layered structure^62^, and the presence of partial A-site deficiency further facilitates the oxygen ion diffusion due to the increase in the oxygen vacancy concentration^24,63^. Given the unchanged contribution from the AEM process, the boosting in electrocatalytic activity through Si incorporation can instead be attributed to the enhanced lattice oxygen participation during the operation of the LOM mechanism.

### Important role of lattice oxygen participation

To further support the conclusion of lattice oxygen participation in enhancing the OER activity, we tested Si-incorporated samples with different intentional doping amounts (i.e., *y* = 0.03, 0.07 and 0.10 in SrCo_1–*y*_Si_*y*_O_3–*δ*_) and evaluated the influence on oxygen diffusion properties and subsequent impact on the OER activity. Contrary to a previous report suggesting that Si can be incorporated up to *y* = 0.07^64^, the actual Si solubility for all our samples was limited to around 3% and the extra amount of Si formed Sr_2_SiO_4_ instead according to Rietveld refinement of the XRD data (Supplementary Fig. 7 and Supplementary Table 2), which is likely due to the difference in synthesis conditions. For simplicity, we nonetheless mark these samples as SCSi0.03, SCSi0.07 and SCSi0.10. While the level of Si incorporated remains similar across these samples, the presence of A-site deficiency in the major perovskite phase associated with the formation of Sr-containing impurities contributed to an increase in oxygen vacancy concentrations with increasing intentional doping amount, both in the bulk and at the surface (Supplementary Tables 5 and 6, and Supplementary Fig. 8). This increase in oxygen vacancy content was also evidenced by the gradual positive shift of oxygen intercalation peaks (Supplementary Fig. 9). However, the oxygen ion diffusivity first experienced an increase to reach a maximum at *y* = 0.05 and then decreased with further increasing *y* to 0.10 (Supplementary Fig. 10 and Supplementary Table 9). This trend in *D*_O_ can be understood from the inhibiting effect of the Sr_2_SiO_4_ impurity for charge transfer. Sr_2_SiO_4_, which is an insulator based on our electrical conductivity tests, shows negligible conductivity (Supplementary Table 10). Based on the EDS mapping (Supplementary Fig. 11), this impurity phase is highly distributed inside the sample, and thus causes a blocking effect for charge transfer across the grains. This hypothesis is supported by the electrical conductivity trend of the various Si-incorporated samples. The pristine SCO shows a conductivity of 2 S cm^−1^ at room temperature, which accords with the literature result^38^. Incorporating Si into the perovskite lattice led to a substantial increase in conductivity by roughly two orders of magnitude for *y* = 0.05 (198 S cm^−1^), beyond which a quick decrease in conductivity was observed for SCSi0.07 and SCSi0.10 (Supplementary Table 10). The change in the lattice structure is a main reason for the substantial increase in electrical conductivity at *y* = 0.05, while the counteracting influence of the Sr_2_SiO_4_ insulating phase leads to the conductivity decrease for *y* > 0.05.

**Fig. 5:**
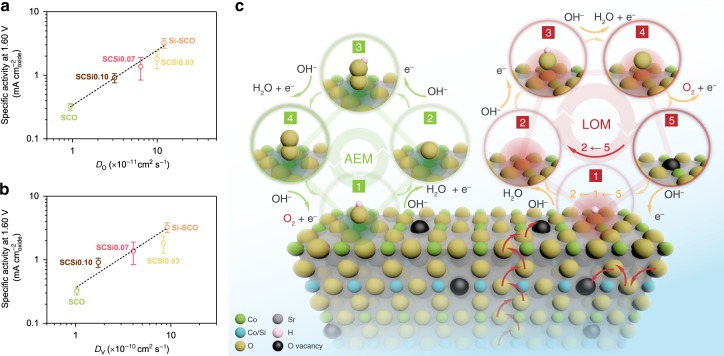
Activity correlations and lattice oxygen participation in the OER on Si-incorporated strontium cobaltites. **a** Correlation of intrinsic OER activity in 0.1 M KOH with the oxygen anion diffusion rate. **b** Correlation of intrinsic OER activity in 0.1 M KOH with the oxygen vacancy diffusion rate. **c** A schematic illustration of the AEM and LOM reaction pathways on Si-incorporated strontium cobaltites. The AEM occurs via concerted proton-coupled electron-transfer steps on the transition metal site, while the LOM operates via non-concerted proton–electron transfer steps involving the participation of lattice oxygen. The mobility of lattice oxygen is also illustrated to highlight its important role in enhancing lattice oxygen participation.

Following the same electrochemical measurements, the OER activity of the various Si-incorporated samples was obtained (Supplementary Fig. 12). Decreased oxygen anion diffusion was found to lead to decreased OER activity for SCSi0.07 and SCSi0.10. Of significance, the intrinsic OER activity correlates strongly with the oxygen ion diffusion rate, as demonstrated in Fig. 5a. We note that the minor impurity phases of Sr_2_Co_2_O_5_^65^ and/or Sr_2_SiO_4_ (Supplementary Fig. 13) contribute negligibly to the observed OER activity. Meanwhile, any activity contribution from the variation of the AEM pathway can be ruled out given the almost identical chemical state of surface Co cations (Supplementary Figs. 14 and 15). It further confirms that the enhancement in OER activity for Si–SCO as compared to SCO is a result from the enhanced lattice oxygen participation, which is directly correlated to the oxygen ion diffusion rate. Since the diffusion of oxygen anions is physically equivalent to that of oxygen vacancies in the opposite direction, the oxygen vacancy diffusion coefficient (*D*_V_) may be calculated using Eq. (5)^66^:

5 $$D_{\mathrm{O}} \cdot c_{\mathrm{O}} = D_{\mathrm{V}} \cdot c_{\mathrm{V}}$$

where *c*_O_ and *c*_V_ are the concentrations for oxygen anions (3 − *δ*) and oxygen vacancies (*δ*), respectively. Applying this conversion, the oxygen vacancy diffusion rate is also found to correlate with the OER activity (Fig. 5b).

## Discussion

As mentioned previously, two mechanisms are currently available for the OER over a perovskite electrocatalyst, i.e., AEM and LOM. The classical AEM mechanism focuses solely on the redox activity of the surface transition metal cations. In this scheme, oxygen products evolve from adsorbed water molecules following concerted proton-coupled electron-transfer steps through four intermediate states^7^ (M–OH, M–O, M–OOH and M–OO, where M denotes the transition metal active site), as shown in Fig. 5c. The binding strength of these intermediate states is found to be strongly correlated to one another^20^, thus imposing a theoretical minimum overpotential on the AEM-based catalysts that cannot be otherwise overcome. The LOM mechanism is different in that it considers the redox of lattice oxygen. As schematically illustrated in Fig. 5c, one possible LOM pathway^25^ involves the participation of five intermediate states, in the sequence of M–**O**H, M–**O**, M–**O**OH, M–**O**O and M–$\square$ (here **O** in bold denotes the oxygen active site and the square box denotes the oxygen vacancy). Despite the similarity in the form of these intermediates to those in the AEM, the LOM differs in the generation of a vacant oxygen site upon the evolution of a lattice oxygen-containing oxygen molecule, which is associated with the decoupling of a certain proton–electron transfer step, giving rise to the previously observed pH-dependent OER kinetics. The lattice oxygen evolved at the surface (which leaves behind a surface vacancy) will be quickly replenished by oxygen ions diffusing from the bulk of the electrocatalyst. The participation of the bulk in the catalysis process thus bypasses the scaling relations dictated in the AEM-based reaction process. Increasing the oxygen ion diffusion rate will facilitate the refilling of the surface lattice oxygen as it is consumed, consequently promoting the catalytic OER process. The participation of lattice oxygen in redox reactions is actually well demonstrated in the field of high-temperature solid oxide fuel cells, in which the introduction of oxygen-ion conductivity into the cathode effectively extends the active sites from the conventional electrolyte-electrode-air triple boundary to the whole electrode surface, thus greatly improving the cathode performance for the oxygen reduction reaction^62,63,67^.

It is also likely that hydroxide ions from the electrolyte can refill the generated oxygen vacancy^25^, which can either provide refreshed oxygen active sites or intercalate into the bulk to compensate for the charge imbalance caused by the previously mentioned oxygen diffusion from the bulk to the surface^68^. With oxygen being the active site, the importance of oxygen ion mobility can be further supported by the hypothesis that it offers the possibility for transporting inactive or less active oxygen to the active oxygen site, thereby allowing increased numbers of oxygen sites to take part in the reaction and consequently boosting the intrinsic catalytic activity. While determination and tracing of such active oxygen sites can be a formidable challenge, our experimental observation does suggest a link between the increase in the intrinsic OER activity and the extent to which the oxygen ion diffusivity is enhanced.

The above discussion thus points to the need for considering a dynamic catalyst surface that has strong interactions not only with the electrolyte but also with the bulk for electrocatalysts operating via the LOM mechanism. However, such dynamics can lead to an unstable surface region, especially in cases of high activity, where the rate of surface oxygen vacancy refilling cannot compete with that of surface vacancy formation (due to fast oxygen evolution), causing the formation of under-coordinated cation sites that become prone to dissolution^49^. This explains the surface amorphization of our Si–SCO catalyst despite its fast oxygen diffusivity associated with the unique A-site deficient layered structure. In fact, the surface reconstruction is recently claimed to be a general trend for perovskite OER catalysts using the LOM mechanism^21,49^. Constructing perovskite surfaces which allow fast enough kinetics for oxygen vacancy refilling appears to be one direct means to address this issue. In another likely solution, control over a constant dissolution/deposition process should be achieved to fulfil the stability requirement, as exemplified by the so-called self-healing mechanism on electrodeposited Co–phosphate catalyst^27^.

In summary, we have demonstrated a model system of Si-incorporated strontium cobaltites, on which the OER occurs with the contribution of the LOM mechanism at different extents that strongly correlates to the oxygen ion diffusivity, a guiding parameter that can be facilely obtained through electrochemical experiments. Our findings not only provide new opportunities to design cost-effective, highly efficient materials for OER catalysis, but also deepen our understanding of the OER mechanisms by which they operate. The next step would be to design more stable perovskite surfaces to further drive advances in water oxidation electrocatalysts applicable for the electrolysis of water, dinitrogen and carbon dioxide.

## Methods

### Materials synthesis

Si-incorporated perovskite samples were prepared by a ball-milling-assisted solid-state reaction approach. Freshly dried chemicals of SrCO_3_, Co_3_O_4_ and SiO_2_ (Sigma-Aldrich) were weighted according to the stoichiometric ratio of SrCo_1–*y*_Si_*y*_O_3–*δ*_ (*y* = 0.00, 0.03, 0.05, 0.07 and 0.10) with different intentional Si-doping levels. The precursory powders were then mixed in an acetone medium for 1 h using a high-energy ball mill (Planetary Mono Mill, Pulverisette 6, Fritisch) at a rotation of 400 rpm. The as-obtained mixtures were dried, pressed into pellets and subjected to calcination in air under ambient pressure at 1000–1200 °C for 24 h with intermediate grindings. The actual composition of each sample after calcination was analysed by XRD (Supplementary Tables 1 and 2).

### Characterisations

XRD data were acquired over a 2*θ* range of 10–80° on a Bruker D8 Advance diffractometer with a copper tube. The phase structures were analysed by Rietveld refinement using the GSAS programme and EXPGUI interface^69^. HR-TEM, HAADF-STEM and EDS mapping were performed using a FEI Titan G2 80-200 TEM/STEM operating at 200 kV. SEM was taken using a Zeiss Neon 40EsB instrument. XPS was conducted on a Kratos Axis Ultra DLD spectrometer with a monochromatic Al Kα irradiation source. The electron binding energy scale was calibrated to the C 1 *s* peak for adventitious carbon, set at 284.8 eV. NEXAFS experiments were performed at the Soft X-ray (SXR) Beamline at the Australian Synchrotron^70^. Spectra were collected using a channeltron detector in partial electron yield mode at a 55° incident angle. Data were analysed using the QANT software package^71^. The photon energy was calibrated by applying the offset required to shift the concurrently measured reference spectra of Co foil (for Co L-edge) to its known energy position. The specific surface area was determined by multipoint Kr adsorption tests under liquid nitrogen temperature (77.3 K) on a Micromeritics TriStar II Plus instrument. Approximately 3.0 g of samples were degassed by heating in vacuum at 200 °C for 5 h prior to each test. The surface area was calculated using the BET equation, assuming that the value for the cross-sectional area of a Kr molecule at liquid nitrogen temperature is 0.210 nm^2^. Electrical conductivity was measured in air at room temperature based on a four-probe DC configuration using a Keithley 2420 source metre. The average bulk Co oxidation state and oxygen vacancy concentration were determined by iodometric titrations.

### Electrochemical measurements

Electrochemical measurements were carried out under ambient conditions using an RDE-based, three-electrode configuration (Pine Research Instrumentation). A catalyst-modified glassy carbon (GC) RDE (0.196 cm^2^), a Pt wire, and a Ag/AgCl (4 M KCl) (all from Pine Research Instrumentation) served as the working, counter and reference electrode, respectively. Prior to use, the GC electrode was polished using Al_2_O_3_ slurries to give a mirror finish and then cleaned by ultrasonication in Milli-Q water (18.2 MΩ cm). The working electrode was prepared by dropcasting 5 µL of an ultra-sonically dispersed catalyst ink, which contains 10 mg of perovskite oxide, 2 mg of Super P® carbon black (Alfa Aesar), 900 μL of absolute ethanol and 100 μL of 5 wt% Nafion® 117 solution (Sigma-Aldrich), onto the GC surface, yielding an approximate catalyst loading of 0.255 mg_oxide_ cm^−2^. The electrolyte was prepared using Milli-Q water and KOH pellets (99.99%, Sigma-Aldrich). O_2_ saturation was maintained to ensure the O_2_/H_2_O equilibrium at 1.23 V vs. RHE. The electrochemical data were collected on a CH Instruments CHI760E potentiostat. To make the catalyst electrochemically accessible, the working electrode, held stationary, was first subjected to CV cycling between −0.6 and −0.2 V vs. Ag/AgCl at 100 mV s^−1^ until a stable CV curve was obtained^72^. Afterwards, the electrocatalytic performance was evaluated by running CV scans at 10 mV s^–1^ with the electrode rotated at 2000 rpm to readily get rid of gaseous O_2_ bubbles evolved at the catalyst surface. To compensate for capacitive effects, the anodic and cathodic scans were averaged. Ohmic losses were corrected by subtracting the ohmic voltage drop from the measured potential using an electrolyte resistance (≈45 Ω) determined by electrochemical impedance spectroscopy. All potentials were reported in the RHE scale, which was converted from the Ag/AgCl reference electrode scale by applying the equation: *E*_RHE_ = *E*_Ag/AgCl_ + 0.199 + 0.0591 × pH (V). The overpotential (*η*), defined as the gap between the applied potential and the equilibrium potential, was calculated based on the equation: *η* = *E*_RHE_ − 1.229 (V). The geometric current density (in mA cm^−2^_geo_) and specific activity (in mA cm^−2^_oxide_) were obtained by normalising the OER current to the geometric surface area of the GC electrode and the BET surface area of the perovskite oxide, respectively. The Tafel plot was constructed using steady-state currents collected from multistep chronoamperometry^73^ in a potential range of 0.6–0.7 V vs. Ag/AgCl at a 10 mV increment, with the ohmic drop compensated for.

### Oxygen intercalation and diffusion coefficient measurements

The electrochemical oxygen intercalation was performed at room temperature in an Ar-saturated 6 M KOH solution using a catalyst-modified GC working electrode, a Pt wire counter electrode, and a Hg/HgO reference electrode. CV was run at a 20 mV s^−1^ scan rate with the working electrode being stationary. To measure the oxygen ion diffusion coefficient, chronoamperometry was performed on the same working electrode by applying a potential 50 mV more anodic of the *E*_1/2_ (defined as the potential halfway between the peak currents for oxygen insertion and extraction). During the chronoamperometry testing, the rotation rate was set at 2000 rpm to remove any electrolyte-based mass-transfer effect. The chronoamperometry data were plotted as current versus the inverse square root of time (*i* vs. *t*^−1/2^), in which the linear portion was fitted to obtain the intercept with the *t*^−1/2^ axis (at *i* = 0). Using a bounded three-dimensional diffusion model^24,56,57^, this intercept was used to calculate the oxygen ion diffusion coefficient according to the equation *λ* = *a*(*D*_O_*t*)^−1/2^, where *λ* is a dimensionless shape factor, *a* is the radius of the particle and *D*_O_ is the diffusion coefficient. Here, *λ* was assumed to be 2, which is representative of a rounded parallelepiped, halfway between the values for a sphere (*λ* = 1.77) and a cube (*λ* = 2.26). *a* was estimated using the relation of *S* = 6/(2*aρ*) based on a spherical geometry approximation, where *S* is the surface area measured from the BET method and *ρ* is the theoretical density determined by Rietveld analysis.

## Supplementary information

Supplementary Information

## Data Availability

The data that support the findings of this study are available from the corresponding author upon reasonable request.
