# Supplementary material for: Direct evidence of boosted oxygen evolution over perovskite by enhanced lattice oxygen participation
Source: Nat Commun. 2020 Apr 24;11:2002. doi: 10.1038/s41467-020-15873-x (PMC7181763; doi:10.1038/s41467-020-15873-x)
Supplement: Supplementary file 1 — Supplementary Information [file 41467_2020_15873_MOESM1_ESM.pdf]

Direct evidence of boosted oxygen evolution over perovskite by enhanced lattice oxygen participation

Pan *et al.*

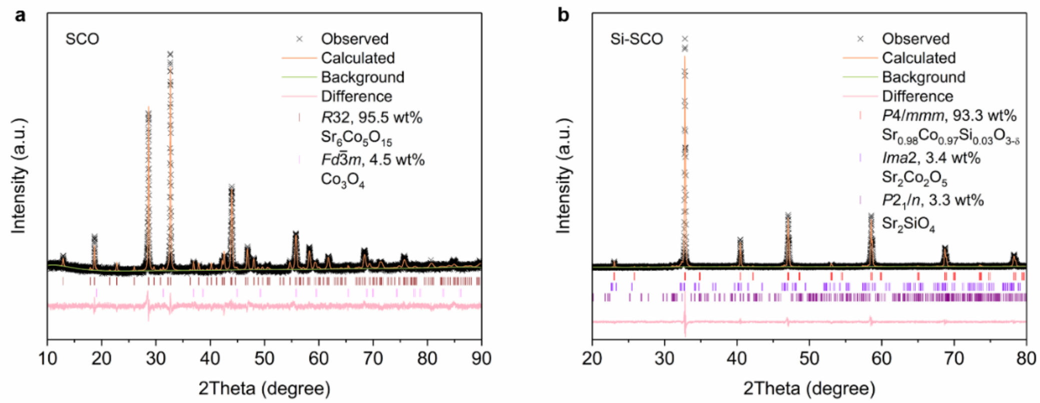

Supplementary Figure 1. Rietveld refinement analysis of XRD patterns. **a** SCO. **b** Si-SCO.

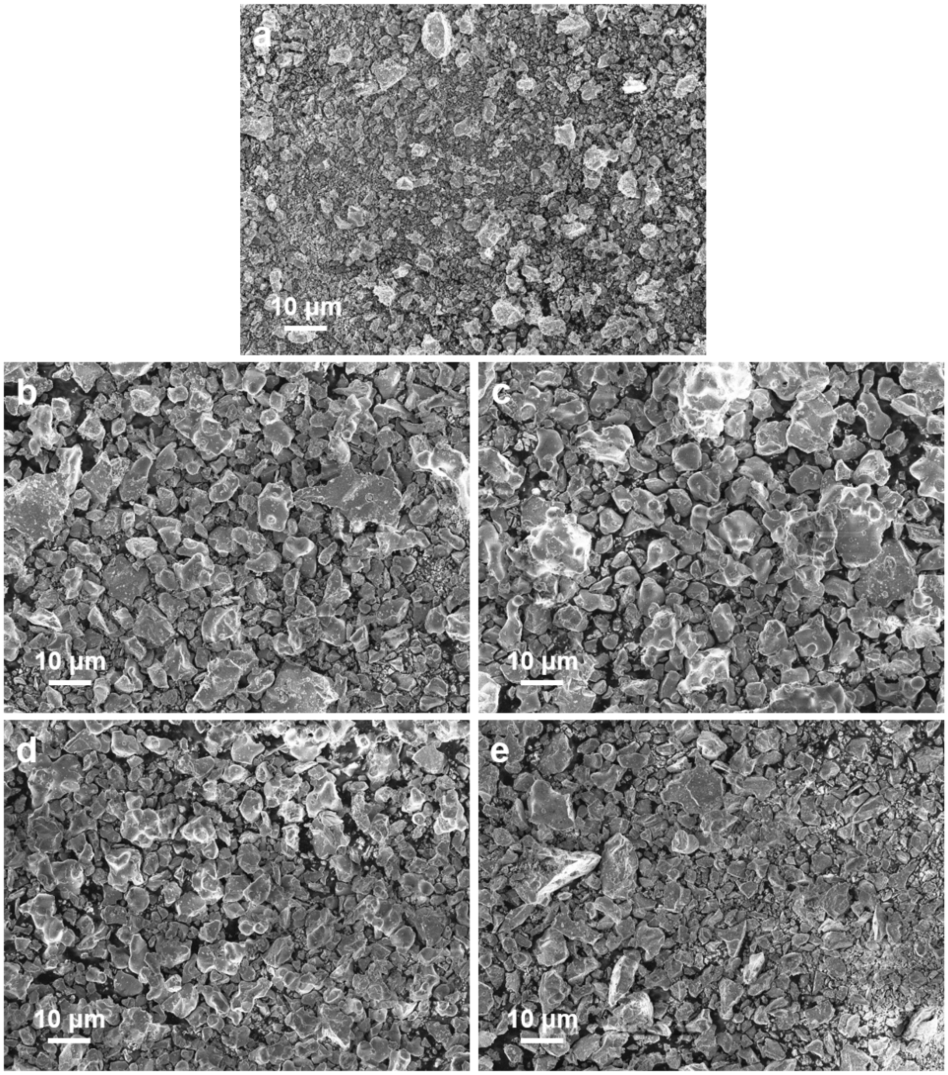

Supplementary Figure 2. SEM images of SCO and Si-incorporated SCO perovskites. **a** SCO. **b** SCSi0.03. **c** Si-SCO. **d** SCSi0.07. **e** SCSi0.10.

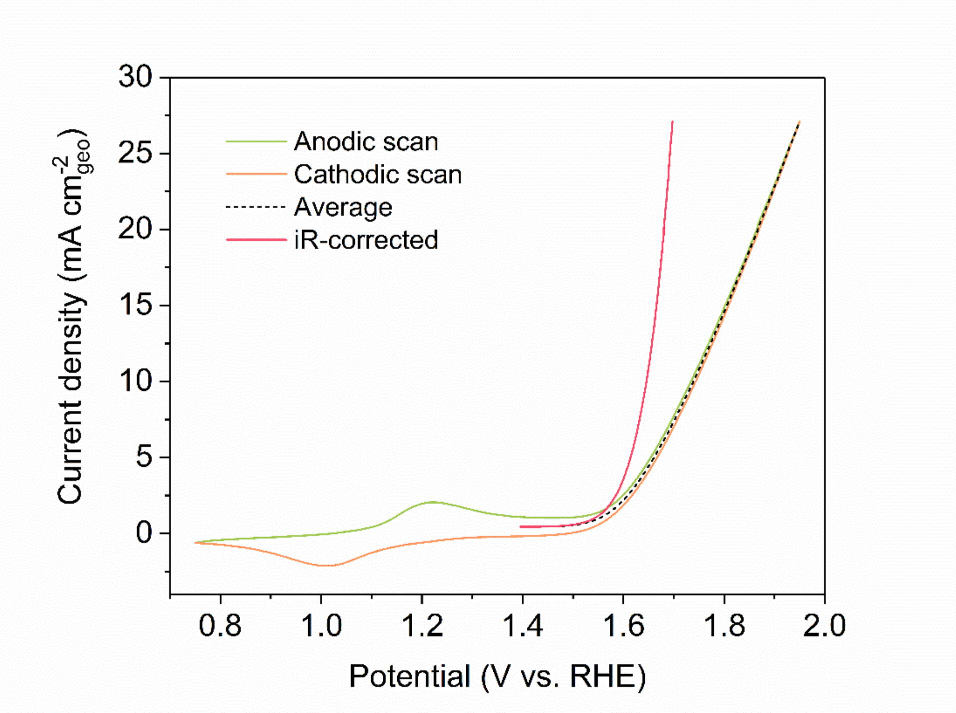

Supplementary Figure 3. Capacitive and ohmic corrections of the CV scans of Si-SCO catalyst to yield the OER kinetic currents. The as-measured OER activity of Si-SCO was first capacity-corrected by averaging the anodic and cathodic scans, and then *iR*-corrected by subtracting the ohmic voltage drop from the measured potential using the electrolyte resistance (≈45 Ω). Due to the presence of redox peaks associated with oxygen intercalation/deintercalation preceding the OER, the corrections here were performed only for CV curves in the OER regime (>1.4 V vs. RHE).

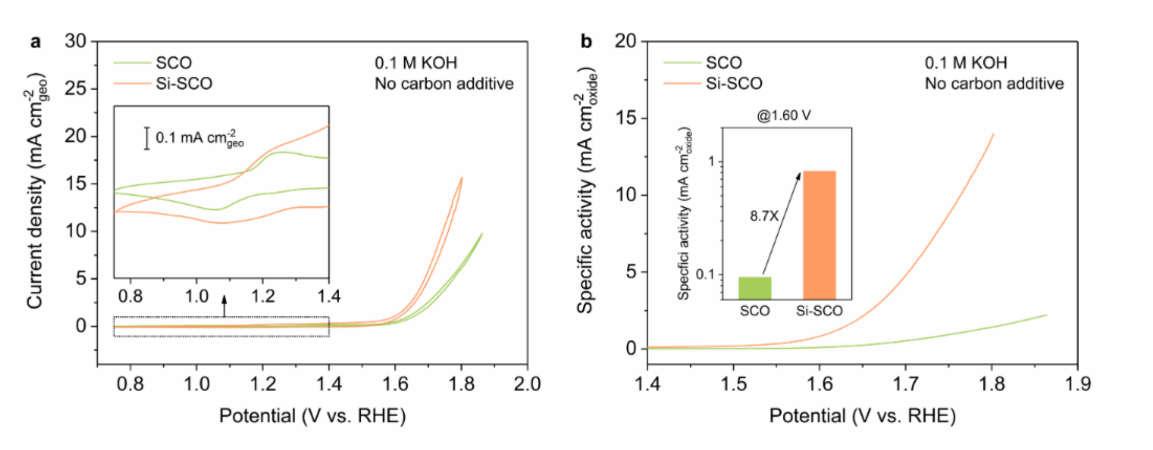

Supplementary Figure 4. Electrocatalytic oxygen evolution performance of SCO and Si-SCO in the absence of conductive carbon. **a** CV scans of SCO and Si-SCO. Inset shows an enlargement at the potential range preceding the OER where redox peaks associated with oxygen intercalation/deintercalation were observed. **b** OER specific activity of SCO and Si-SCO. Inset shows a comparison of specific activity at 1.60 V vs. RHE. All measurements were conducted in an O_2-saturated 0.1 M KOH electrolyte under ambient conditions.

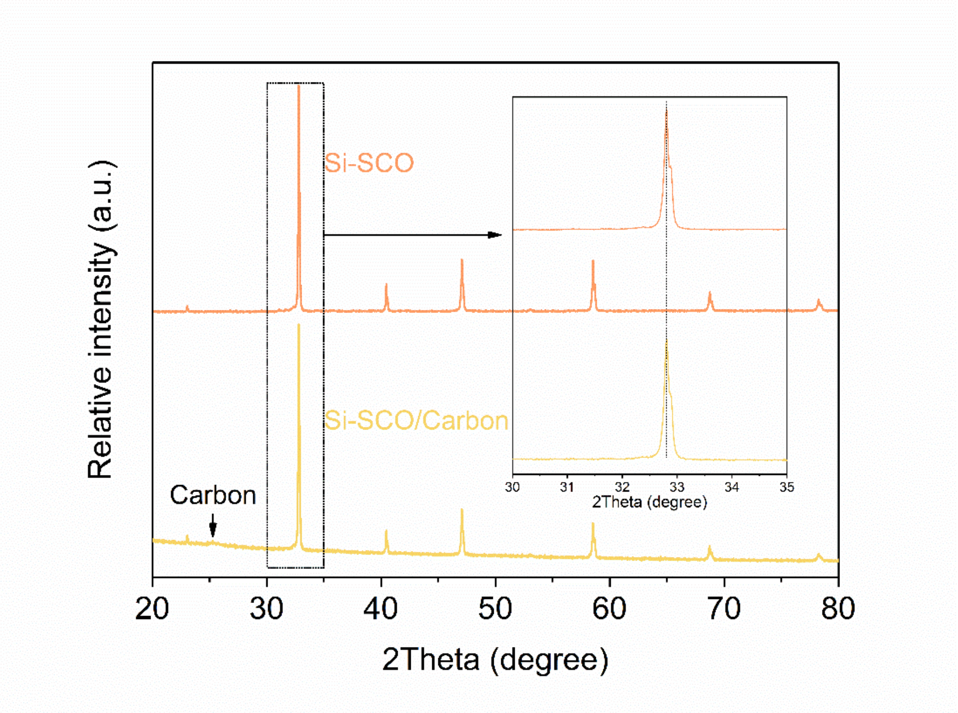

Supplementary Figure 5. XRD patterns for Si-SCO and Si-SCO/carbon composite. Inset shows no obvious shift of the main diffraction peak, suggesting that the electronic structure of Si-SCO remains intact after physically mixed with conductive carbon.

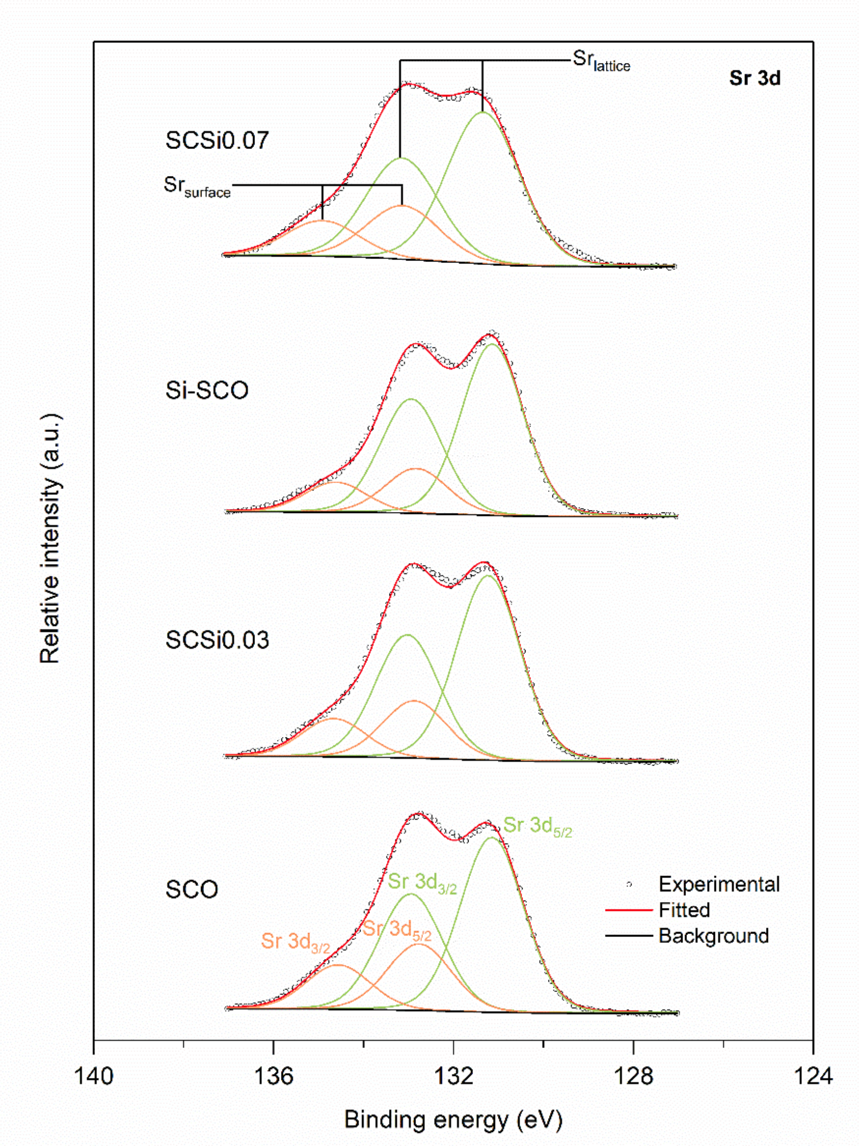

Supplementary Figure 6. Sr 3*d* core-level XPS spectra of SCO and Si-incorporated SCO perovskites, with peak fitting results based on two components, i.e., lattice Sr ($Sr_{lattice}$) at a lower binding energy and surface Sr ($Sr_{surface}$) at a higher binding energy.

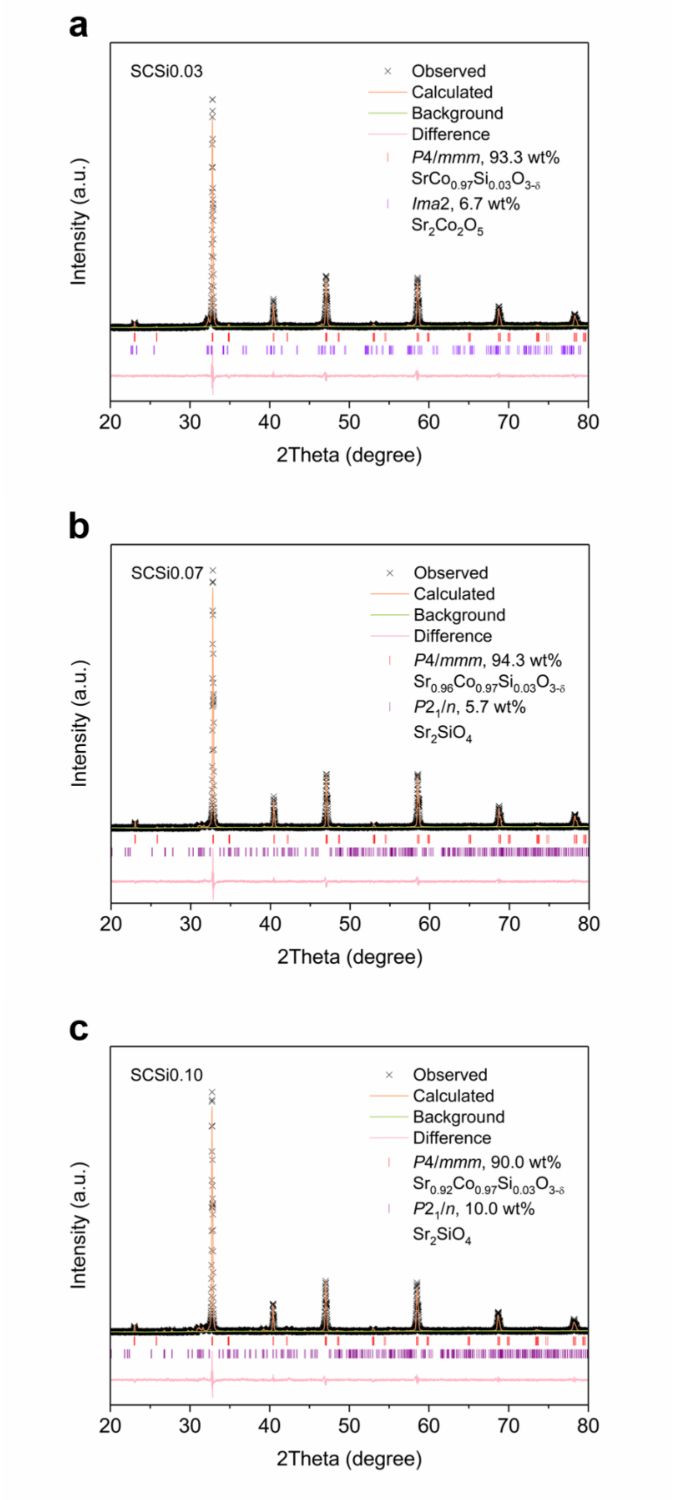

Supplementary Figure 7. Rietveld refinement analysis of the XRD patterns of Si-incorporated SCO perovskites. **a** SCSi0.03. **b** SCSi0.07. **c** SCSi0.10.

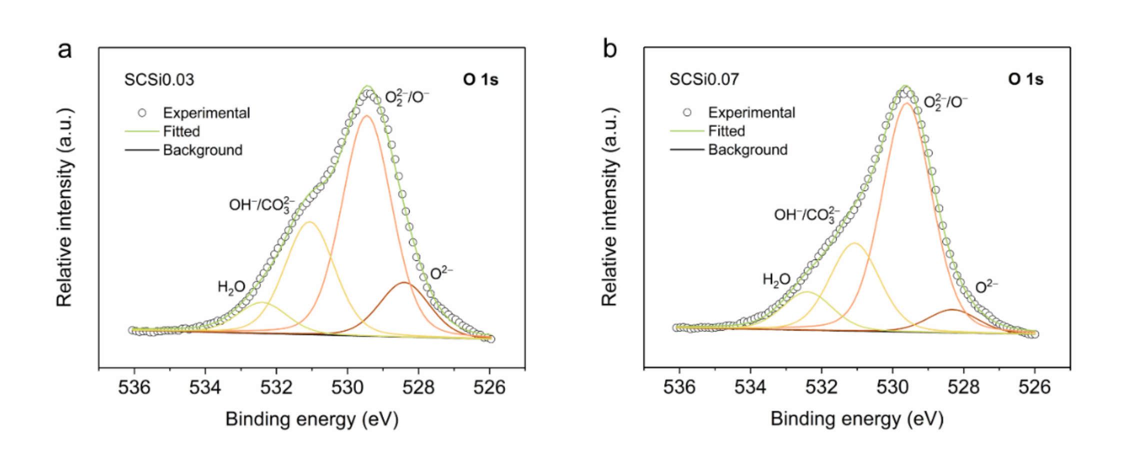

Supplementary Figure 8. O 1*s* core-level XPS spectra of Si-incorporated SCO perovskites. **a** SCSi0.03. **b** SCSi0.07.

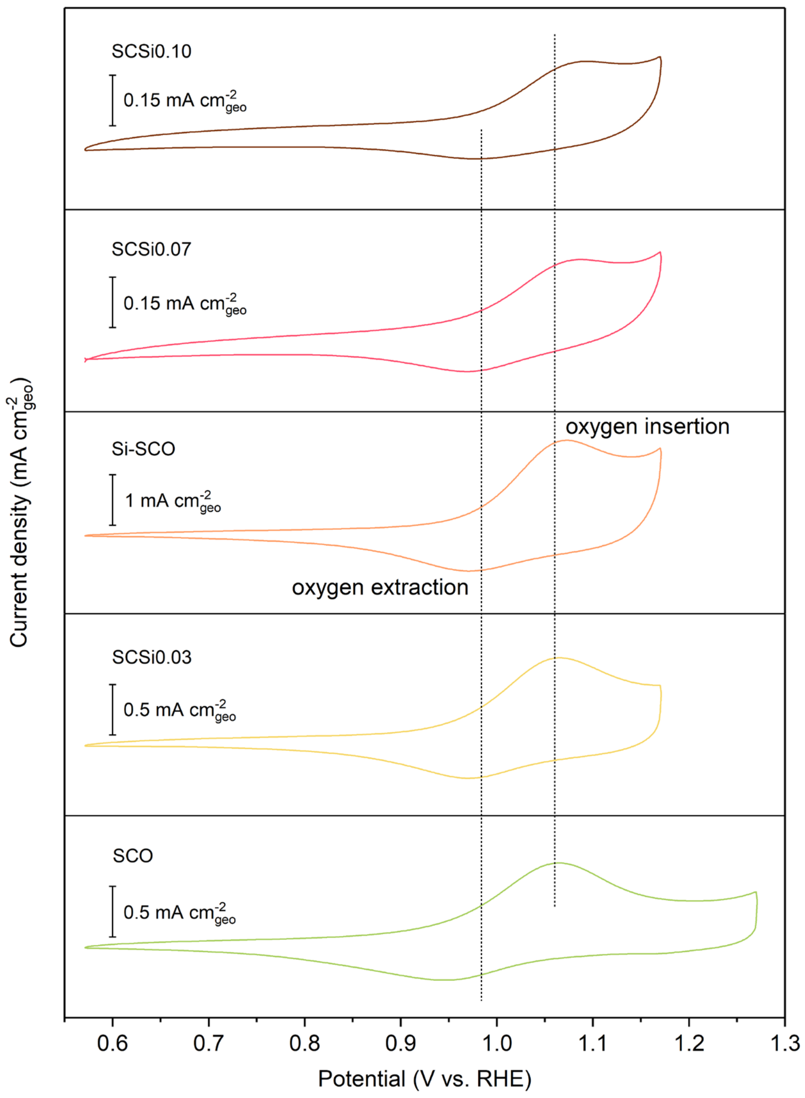

Supplementary Figure 9. CV curves of SCO and Si-incorporated SCO perovskites in Ar-saturated 6 M KOH. The redox peaks indicate the electrochemical intercalation of oxygen. Overall, a positive shift of the redox peaks was observed with the increasing intentional doping amount of Si, indicative of an increase in oxygen vacancy content.

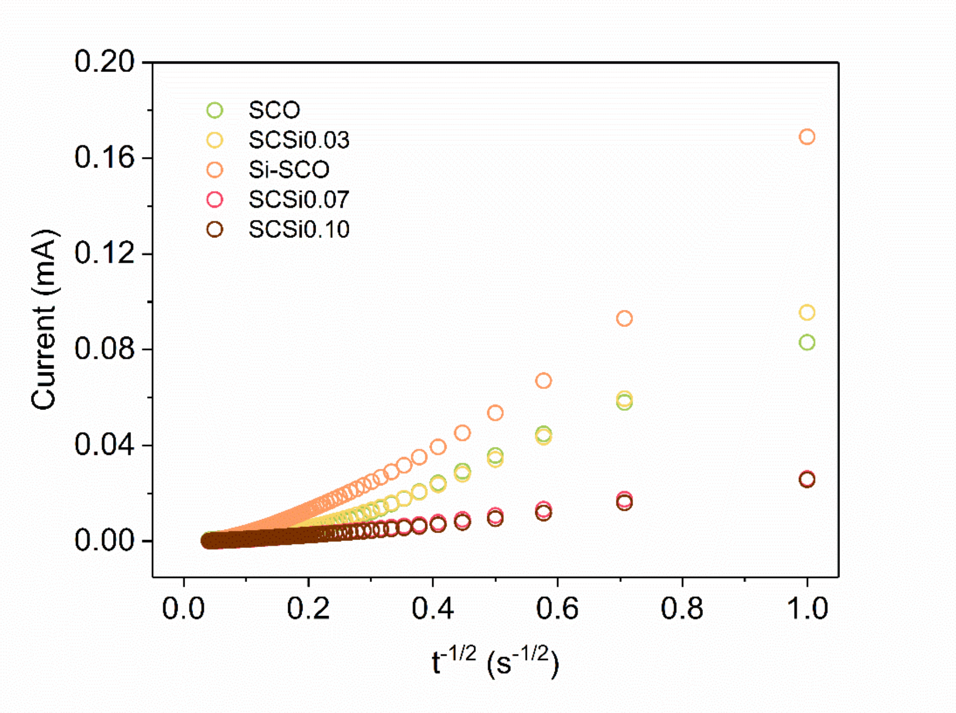

Supplementary Figure 10. Chronoamperometry data (current vs. $t^{-1/2}$) of SCO and Si-incorporated SCO perovskites used for the calculation of oxygen ion diffusion coefficients.

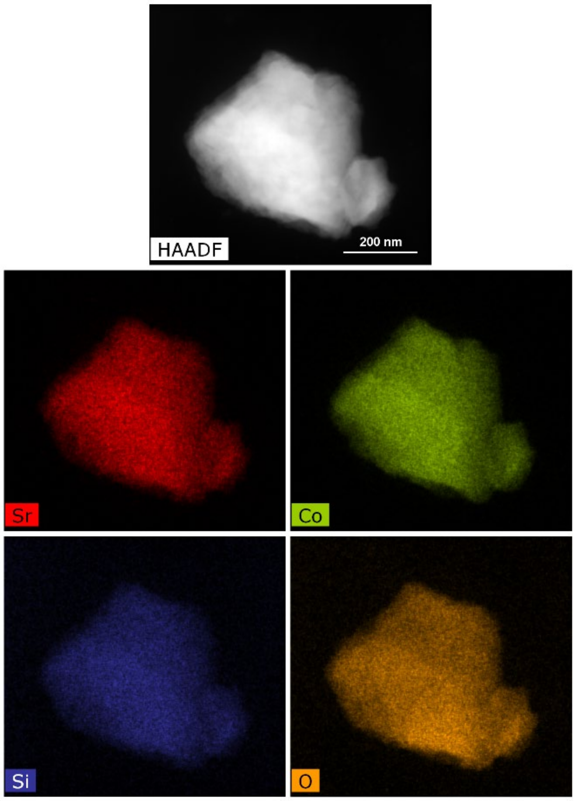

Supplementary Figure 11. HAADF-STEM image of SCSi0.10 and the corresponding EDS mapping images of Sr, Co, Si, and O.

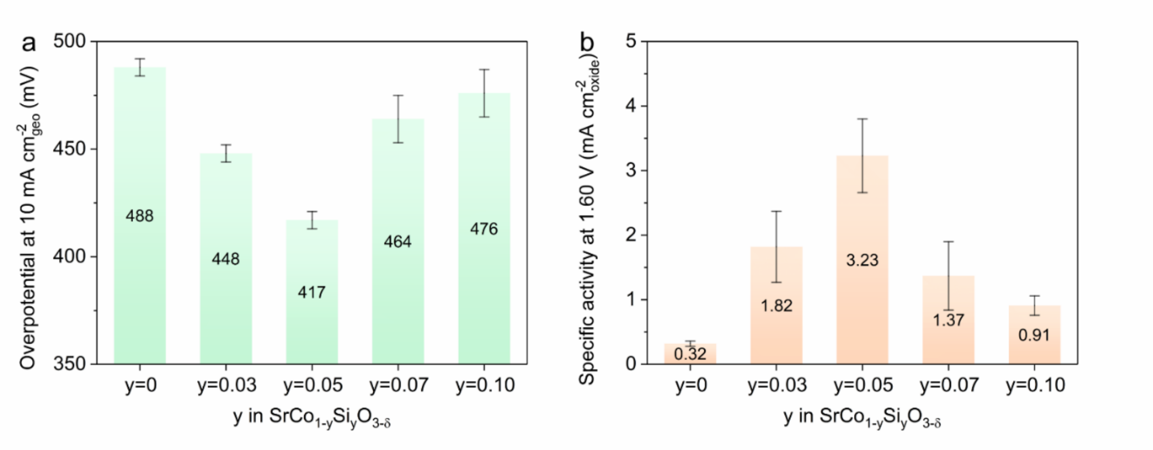

Supplementary Figure 12. Comparison of the OER activity of $SrCo_{1-y}Si_yO_{3-\delta}$. **a** Overpotential needed to afford a 10 mA cm$^{-2}_{geo}$ current density. **b** Specific activity at 1.60 V vs. RHE. The electrochemical measurements were conducted in an O_2-saturated 0.1 M KOH electrolyte. Error bars are the standard deviations of triplicate measurements.

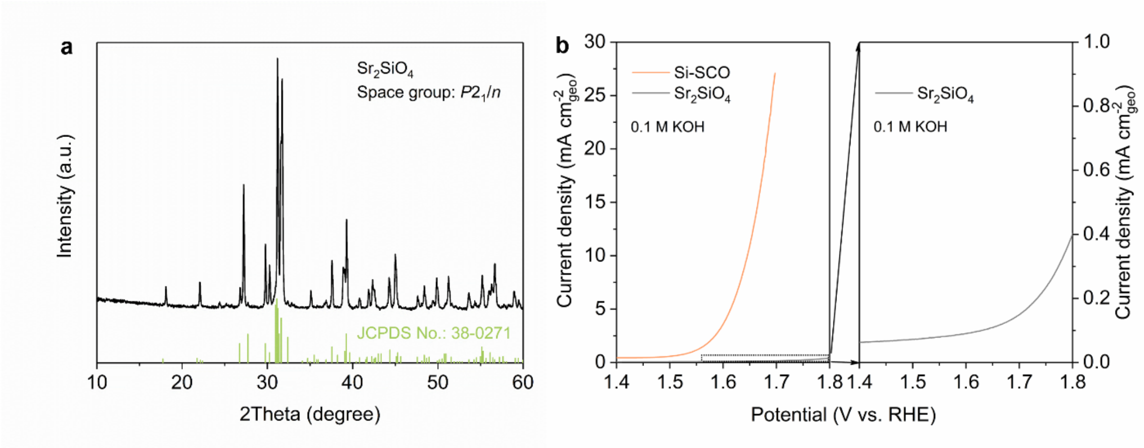

Supplementary Figure 13. Characterization of synthesized Sr_2SiO_4 and its OER activity. **a** XRD pattern of Sr_2SiO_4 synthesized from solid-state reaction of $SrCO_3$ and SiO_2 at 1100 °C in air. The standard XRD peaks of Sr_2SiO_4 (JCPDS No.: 38-0271) are shown for reference. **b** OER kinetic currents of Sr_2SiO_4 in an O_2-saturated 0.1 M KOH electrolyte, showing negligible OER activity.

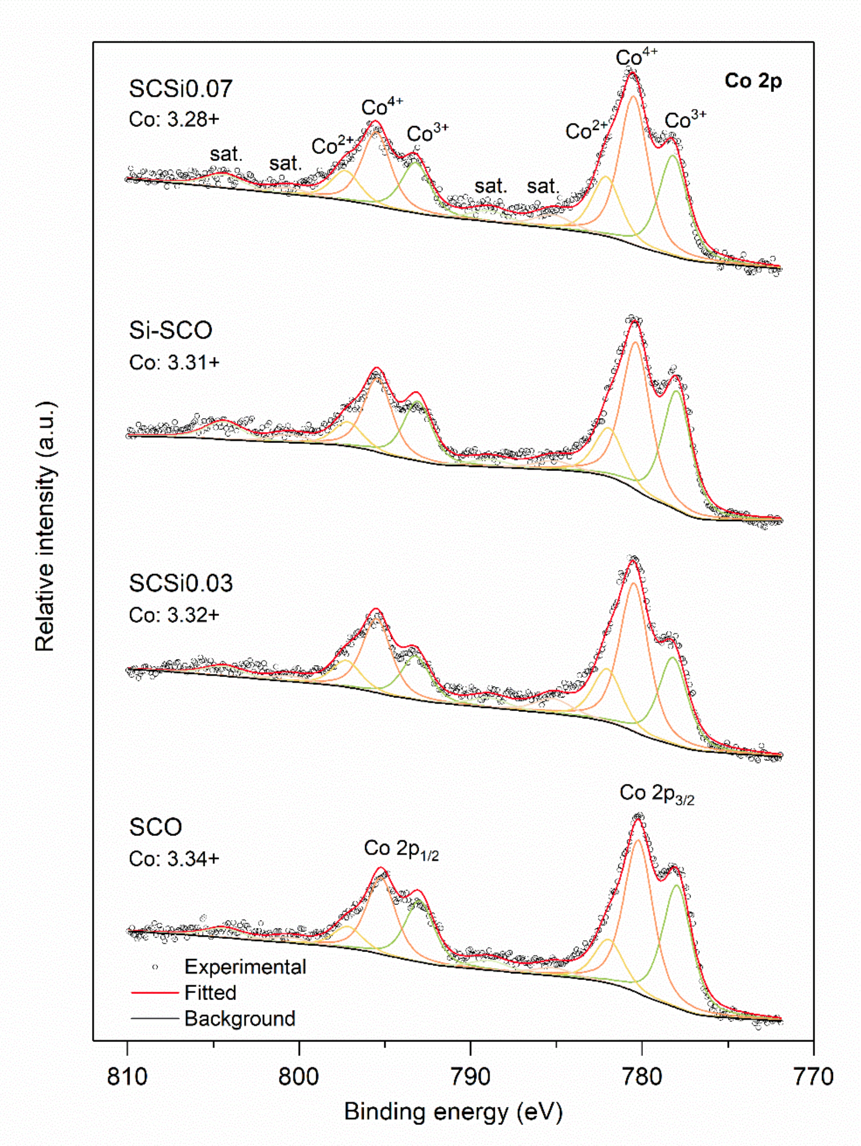

Supplementary Figure 14. Co 2*p* core-level XPS spectra of SCO and Si-incorporated SCO perovskites, with peak fitting results based on multiple cobalt species. Here sat. denotes satellite peaks.

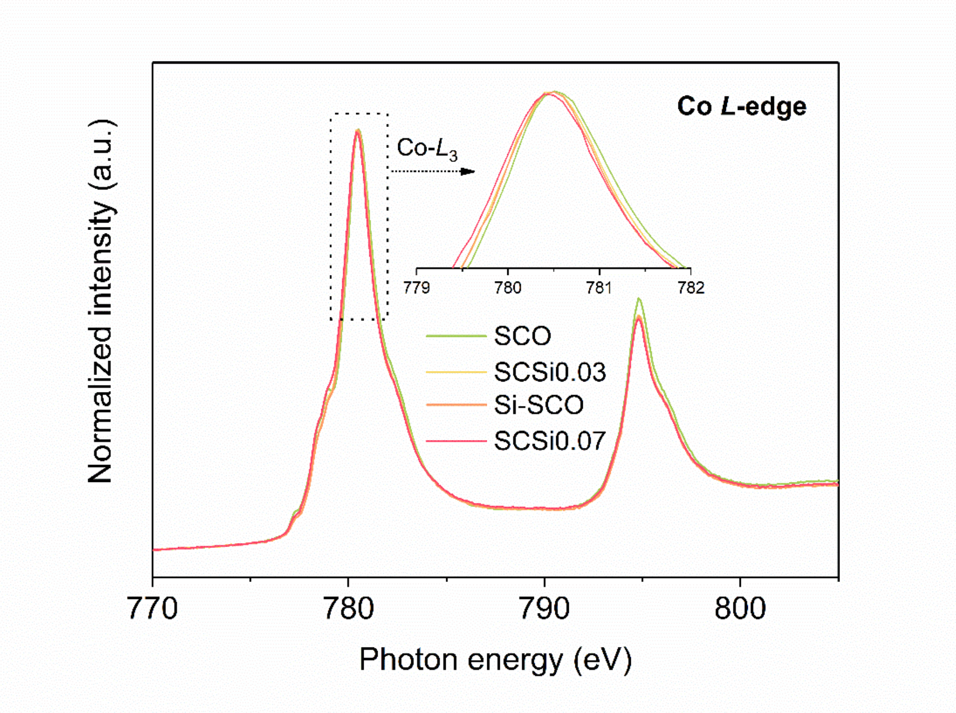

Supplementary Figure 15. Co L-edge NEXAFS spectra of SCO and Si-incorporated SCO perovskites. Inset shows the Co L_3-edge spectra in an expanded photon energy scale.

Supplementary Table 1. Rietveld refinement results of the XRD pattern of SCO.

Phase	Weight percentage	Lattice parameters	Goodness of fit
$Sr_6Co_5O_{15}$ ($R32$)	95.5 wt%	a=b=9.4974(3) Å, c=12.3974(5) Å<br>α=β=90°, γ=120°	R_p=1.57%<br>R_{wp}=2.09%<br>χ^2=1.874
Co_3O_4 ($Fd\bar{3}m$)	4.5 wt%	a=b=c=8.0691(7) Å<br>α=β=γ=90°	

Supplementary Table 2. Rietveld refinement results of XRD patterns of Si-incorporated perovskites.

Perovskite	SCSi0.03	Si-SCO	SCSi0.07	SCSi0.10
Phase compositions [Weight percentage (wt%) and lattice parameters (Å)]				
Tetragonal phase	93.3 wt%	93.3 wt%	94.3 wt%	90.0 wt%
($Sr_{1-x}Co_{1-y}Si_yO_{3-\delta}$) *P*4/*mmm*	a=3.85758(6)<br>c=7.7247(5)<br>α=β=γ=90°	a=3.85917(4)<br>c=7.7270(1)<br>α=β=γ=90°	a=3.86070(4)<br>c=7.7298(1)<br>α=β=γ=90°	a=3.86180(5)<br>c=7.7329(1)<br>α=β=γ=90°
Nominal composition	$SrCo_{0.97}Si_{0.03}O_{3-\delta}$	$Sr_{0.98}Co_{0.97}Si_{0.03}O_{3-\delta}$	$Sr_{0.96}Co_{0.97}Si_{0.03}O_{3-\delta}$	$Sr_{0.92}Co_{0.97}Si_{0.03}O_{3-\delta}$
Brownmillerite phase	6.7 wt%	3.4 wt%	0.0 wt%	0.0 wt%
($Sr_2Co_2O_5$) *Ima*2	a=15.735(2)<br>b=5.5705(8)<br>c=5.4685(8)<br>α=β=γ=90°	a=15.710(3)<br>b=5.568(1)<br>c=5.473(1)<br>α=β=γ=90°		
Monoclinic phase	0.0 wt%	3.3 wt%	5.7 wt%	10.0 wt%
(Sr_2SiO_4) *P*2₁/*n*		a=5.658(1)<br>b=7.073(2)<br>c=9.749(4)<br>α=γ=90° β=92.67(4)°	a=5.659(1)<br>b=7.080(1)<br>c=9.751(2)<br>α=γ=90° β=92.57(2)°	a=5.6621(6)<br>b=7.0821(8)<br>c=9.752(1)<br>α=γ=90° β=92.58(1)°
Reliability factors				
R_p (%)	2.19	2.19	2.16	2.17
R_{wp} (%)	1.52	1.50	1.52	1.56
χ^2	2.243	2.250	2.086	2.140

Supplementary Table 3. BET surface areas of SCO and Si-incorporated SCO perovskites.

Perovskite	Surface area [$m^2\ g^{-1}$]
SCO	1.74
SCSi0.03	0.45
Si-SCO	0.44
SCSi0.07	0.40
SCSi0.10	0.52

Supplementary Table 4. OER activity comparison of Si-incorporated Si-SCO with other perovskite catalysts in 0.1 M KOH.

Perovskite catalyst	Catalyst loading [mg_{oxide} cm^{-2}]	Specific activity @ 1.60 V vs. RHE [mA $cm^{-2}{}_{oxide}$]	η_{10} [mV]	Ref.
Si-SCO	0.255	3.23	417	This work
BSCF[a)] (sol−gel, 1000 °C)	0.232	0.86	~450	[1]
BSCF (sol−gel, 1050 °C)	0.639	~0.7	~550	[2]
BSCF (polymerization, 1100 °C)	0.250	~0.6	NA	[3]
BSCF (solution combustion, 1100 °C)	0.250	~10	NA	[4]
BSCF (solution combustion, 1100 °C)	0.250	~3.5	NA	[5]
PBC[b)] (solid-state reaction, 1100 °C)	0.250	~7.0	NA	[5]
PBC (Pechini process, 1100 °C)	0.202	~0.6	~520	[6]
PBC (sol−gel, 1000 °C)	0.361	~0.2	517	[7]
$LaNiO_3$	0.232	~0.24[c)]	~630	[8]
$LaCuO_3$	0.255	~0.03	NA	[9]
$SrNb_{0.1}Co_{0.7}Fe_{0.2}O_{3-\delta}$	0.232	NA	500	[10]
$BaCo_{0.7}Fe_{0.2}Sn_{0.1}O_{3-\delta}$	0.232	~1.0	~420	[11]
$NdBa_{0.25}Sr_{0.75}Co_2O_{5.9}$	0.800[d)]	NA	470	[12]
	0.800[e)]	NA	420	[12]
$LaCoO_3$ (bulk)	0.250	~0.5[f)]	620	[13]
$LaCoO_3$ (80 nm)	0.250	~0.9[f)]	490	[13]
$La_{0.5}Sr_{0.5}Co_{0.8}Fe_{0.2}O_{3-\delta}$	0.200	NA	~490	[14]
$LaFeO_3$	0.232	0.061[g)]	510	[15]
$CaMnO_{2.5}$	0.255	NA	>500	[16]

[a)] BSCF is the abbreviation for $Ba_{0.5}Sr_{0.5}Co_{0.8}Fe_{0.2}O_{3-\delta}$.

[b)] PBC is the abbreviation for $PrBaCo_2O_{5+\delta}$.

[c)] Obtained at a potential of 1.68 V vs. RHE.

[d)] Loading is based on the composite of perovskite and carbon black.

[e)] Loading is based on the composite of perovskite, carbon black, and polypyrrole.

[f)] Obtained at a potential of 1.72 V vs. RHE.

[g)] Obtained at a potential of 1.63 V vs. RHE.

Supplementary Table 5. The bulk Co oxidation state and oxygen vacancy concentration (*δ*) of SCO and Si-incorporated SCO perovskites, as determined from the iodometric titration method taking consideration into the presence of impurity phases.

Perovskite	Nominal composition of major phase ($Sr_{1-x}Co_{1-y}Si_yO_{3-\delta}$)	Bulk Co oxidation state	*δ*
SCO	$SrCoO_{3-\delta}$	3.50±0.10	0.25±0.05
SCSi0.03	$SrCo_{0.97}Si_{0.03}O_{3-\delta}$	3.34±0.02	0.32±0.01
Si-SCO	$Sr_{0.98}Co_{0.97}Si_{0.03}O_{3-\delta}$	3.32±0.04	0.35±0.02
SCSi0.07	$Sr_{0.96}Co_{0.97}Si_{0.03}O_{3-\delta}$	3.24±0.04	0.41±0.02
SCSi0.10	$Sr_{0.92}Co_{0.97}Si_{0.03}O_{3-\delta}$	3.21±0.02	0.46±0.01

Supplementary Table 6. XPS peak fitting results of O 1*s* core levels of SCO and Si-incorporated SCO perovskites.

Perovskite	O^{2-} [%]	O_2^{2-}/O^- [%]	OH^-/CO_3^{2-} [%]	H_2O [%]
SCO	2	39	51	8
SCSi0.03	13	53	27	7
Si-SCO	13	56	24	7
SCSi0.07	6	61	23	10

Supplementary Table 7. The elemental surface compositions of SCO and Si-incorporated SCO perovskites, as derived from XPS data, taking Co 3*p*% + Sr 3*d*% = 100%.[a)]

Perovskite	Co 3*p* [%]	Sr 3*d* [%]
SCO	30	70
SCSi0.03	26	74
Si-SCO	28	72
SCSi0.07	22	78

[a)] Note that the overlap between Si 2*p* and Co 3*s* limits us from identifying the exact compositions of surface Si. The measured surface composition of Co is around 20–30% for all the samples whereas that of Sr is around 70–80%. This deviates from their stoichiometric values, approximately 50%/50%, indicative of segregation of Sr toward the catalyst surface.

Supplementary Table 8. The $Sr_{surface}$ to $Sr_{lattice}$ ratio ($Sr_{surface}/Sr_{lattice}$) of SCO and Si-incorporated SCO perovskites, as derived from peak deconvolution of Sr 3*d* core-level XPS spectra.

Perovskite	$Sr_{surface}/Sr_{lattice}$
SCO	38
SCSi0.03	31
Si-SCO	26
SCSi0.07	35

Supplementary Table 9. Oxygen ion diffusion coefficient (D_O) and oxygen vacancy diffusion coefficient (D_V) of SCO and Si-incorporated SCO perovskites.

Perovskite	D_O [$\times 10^{-11}$ cm^2 s^{-1}]	D_V [$\times 10^{-10}$ cm^2 s^{-1}]
SCO	0.94	1.03
SCSi0.03	9.96	8.34
Si-SCO	12.04	9.12
SCSi0.07	6.40	4.04
SCSi0.10	3.14	1.73

Supplementary Table 10. Electrical conductivity of SCO, Si-incorporated SCO perovskites, and Sr_2SiO_4 measured in air atmosphere at room temperature.

Material	Electrical conductivity [S cm^{-1}]
SCO	2
SCSi0.03	182
Si-SCO	198
SCSi0.07	46
SCSi0.10	97
Sr_2SiO_4	3×10^{-6}

Supplementary Note 1

The inclusion of conductive carbon in evaluating perovskite oxide electrocatalysts

For the OER activity evaluation, we physically mixed our perovskite oxide catalysts with conductive carbon (as-received Super P® carbon black from Alfa Aesar) to facilitate electrical contact between catalyst particles as well as between the catalyst and the RDE, a practice that has been widely observed for fundamental research across different research groups.[1−12,15,16] We note that the carbon introduced here, especially that without functionalization (e.g., without heteroatom doping), contributes negligibly to the OER performance (**Fig. 2a** of the main text) because it is only weakly coupled to perovskites through physical mixing. We nonetheless tested our catalysts without the inclusion of carbon (**Supplementary Figure 4**). Compared to data presented in **Fig. 2a** of the main text, much lower current densities were found as a result of the poor contact between the catalyst particles due to the absence of conductive carbon. Importantly, a similar increase in intrinsic activity was observed for Si-SCO as compared with SCO. Note that an analogous redox feature associated with oxygen intercalation is also observed for samples without carbon addition, indicating that this feature is inherent to the perovskite oxide catalysts studied here. Additionally, concerns might arise with respect to the change of transition metal electronic state after the introduction of carbon. For example, the oxidation state of cobalt in BSCF was found to be reduced after physically mixed with acetylene black, with evidence from a main peak shift in the XRD patterns[17]. Similarly, we compared the XRD patterns of Si-SCO samples before and after mixing with carbon through ultrasonication (i.e., following exactly the catalyst preparation procedure). As shown in **Supplementary Figure 5**, no shift in XRD main peak was observed, which indicates that strong electronic interaction between perovskite and carbon is absent in our case. We suppose that the different tolerance against reductive carbon is likely correlated with the structural stability and the electronic binding environment of the Co cations.

Supplementary Note 2

The effect of surface Sr segregation on the OER performance of perovskite electrocatalysts

The surface segregation of Sr in the form of carbonate and/or hydroxide (as seen in the O 1*s* core-level XPS results) is often observed in Sr-containing perovskite oxides prepared from conventional methods (e.g., solid-state reaction, sol–gel process), due likely to the high reactivity of Sr cations with the environment[18]. The Sr segregation phenomenon in our Si-incorporated perovskites was further supported by the relatively lower surface Co to Sr ratios (**Supplementary Table 7**) and the presence of surface Sr species in Sr 3*d* core-level XPS spectra (**Supplementary Figure 6** and **Supplementary Table 8**). As seen from these results, no significant difference was found in the surface Co/Sr compositions and surface Sr contents, indicating that a similar extent of Sr segregation is observed across all the samples. We therefore assume that the surface Sr segregation should not play a major role in promoting or inhibiting the OER activity.

Additionally, it is interesting to note that while Sr segregation is considered detrimental in high-temperature electrocatalysis such as solid oxide fuel cell cathodes[19], it does not necessarily make the surface inactive for low-temperature electrocatalysis. For example, Skinner et al. found that Sr doping into the A-site of $GdBaCo_2O_{6-\delta}$ double perovskite caused the segregation of Sr to the sample surface, but interestingly they observed higher OER activity with increasing Sr dopant level[20]. Overall, the effect of Sr segregation on low-temperature OER electrocatalysis represents an interesting research topic, but currently remains largely underexplored, and is mostly neglected in published research based on Sr-containing perovskites. We note, however, that a more detailed investigation into Sr segregation would be beyond the scope of our current manuscript, which mainly focuses on lattice-oxygen participation boosted OER electrocatalysis. We also caution that for future research such Sr segregation effect should

be minimized when investigating other influencing parameters using Sr-containing perovskites, which can be partially done by selecting a suitable synthesis technique.

Supplementary References

1. Xu, X. et al. Toward enhanced oxygen evolution on perovskite oxides synthesized from different approaches: A case study of $Ba_{0.5}Sr_{0.5}Co_{0.8}Fe_{0.2}O_{3-\delta}$. *Electrochim. Acta* **219**, 553–559 (2016).
2. Jung, J.-I. et al. Fabrication of $Ba_{0.5}Sr_{0.5}Co_{0.8}Fe_{0.2}O_{3-\delta}$ catalysts with enhanced electrochemical performance by removing an inherent heterogeneous surface film layer. *Adv. Mater.* **27**, 266–271 (2015).
3. Yagi, S. et al. Covalency-reinforced oxygen evolution reaction catalyst. *Nat. Commun.* **6**, 8249 (2015).
4. Suntivich, J., May, K. J., Gasteiger, H. A., Goodenough, J. B. & Shao-Horn, Y. A perovskite oxide optimized for oxygen evolution catalysis from molecular orbital principles. *Science* **334**, 1383–1385 (2011).
5. Grimaud, A. et al. Double perovskites as a family of highly active catalysts for oxygen evolution in alkaline solution. *Nat. Commun.* **4**, 2439 (2013).
6. Zhao, B. et al. A tailored double perovskite nanofiber catalyst enables ultrafast oxygen evolution. *Nat. Commun.* **8**, 14586 (2017).
7. Sun, H. et al. B-site cation ordered double perovskites as efficient and stable electrocatalysts for oxygen evolution reaction. *Chem. Eur. J.* **23**, 5722–5728 (2017).
8. Yu, J. et al. Activity and stability of Ruddlesden–Popper-type $La_{n+1}Ni_nO_{3n+1}$ (n=1, 2, 3, and ∞) electrocatalysts for oxygen reduction and evolution reactions in alkaline media. *Chem. Eur. J.* **22**, 2719–2727 (2016).
9. Yamada, I. et al. Systematic study of descriptors for oxygen evolution reaction catalysis in perovskite oxides. *J. Phys. Chem. C* **122**, 27885–27892 (2018).
10. Zhu, Y. et al. $SrNb_{0.1}Co_{0.7}Fe_{0.2}O_{3-\delta}$ perovskite as a next-generation electrocatalyst for oxygen

evolution in alkaline solution. *Angew. Chem. Int. Ed.* **54**, 3897–3901 (2015).

11. Xu, X. et al. Co-doping strategy for developing perovskite oxides as highly efficient electrocatalysts for oxygen evolution reaction. *Adv. Sci.* **3**, 1500187 (2016).

12. Lee, D.-G. et al. Polypyrrole-assisted oxygen electrocatalysis on perovskite oxides. *Energy Environ. Sci.* **10**, 523–527 (2017).

13. Zhou, S. et al. Engineering electrocatalytic activity in nanosized perovskite cobaltite through surface spin-state transition. *Nat. Commun.* **7**, 11510 (2016).

14. Park, H. W. et al. Electrospun porous nanorod perovskite oxide/nitrogen-doped graphene composite as a bi-functional catalyst for metal air batteries. *Nano Energy* **10**, 192–200 (2014).

15. Zhu, Y. et al. Enhancing electrocatalytic activity of perovskite oxides by tuning cation deficiency for oxygen reduction and evolution reactions. *Chem. Mater.* **28**, 1691–1697 (2016).

16. Kim, J., Yin, X., Tsao, K.-C., Fang, S. & Yang, H. $Ca_2Mn_2O_5$ as oxygen-deficient perovskite electrocatalyst for oxygen evolution reaction. *J. Am. Chem. Soc.* **136**, 14646–14649 (2014).

17. Fabbri, E., Nachtegaal, M., Cheng X. & Schmidt, T. J. Superior bifunctional electrocatalytic activity of $Ba_{0.5}Sr_{0.5}Co_{0.8}Fe_{0.2}O_{3-\delta}$/carbon composite electrodes: Insight into the local electronic structure. *Adv. Energy Mater.* **5**, 1402033 (2015).

18. Cheng X. et al. Oxygen evolution reaction on $La_{1-x}Sr_xCoO_3$ perovskites: A combined experimental and theoretical study of their structural, electronic, and electrochemical properties. *Chem. Mater.* **27**, 7662–7672 (2015).

19. Druce, J., Téllez, H. & Hyodo, J. Surface segregation and poisoning in materials for low-temperature SOFCs. *MRS Bull.* **39**, 810–815 (2014).

20. Pramana, S. S. et al. Crystal structure and surface characteristics of Sr-doped $GdBaCo_2O_{6-\delta}$ double perovskites: oxygen evolution reaction and conductivity. *J. Mater. Chem. A* **6**, 5335–5345 (2018).
